# Experimental and Theoretical Insights on the Use of Expired Furosemide as Corrosion Inhibition for Cu in NaCl

**DOI:** 10.3390/ma19153274

**Published:** 2026-08-03

**Authors:** Dalia Garcia-Rosas, Alfredo Brito-Franco, Hugo Albeiro Saldarriaga-Noreña, Roy Lopez-Sesenes, America Maria Ramirez-Arteaga, Ana Karen Galvez-Larios, Jesus Porcayo-Calderon, Jose Gonzalo Gonzalez-Rodriguez

**Affiliations:** 1 Facultad de Ciencias Quimicas e Ingenieria, Universidad Autonoma del Estado de Morelos, Avenida Universidad 1001, Col. Chamilpa, Cuernavaca 62209, Mexico; dalia.garciaros@fcqei.uaem.edu.mx (D.G.-R.); rlopez@uaem.mx (R.L.-S.); america.rmz@uaem.mx (A.M.R.-A.); 2 Centro de Investigacion en Ingenieria y Ciencias Aplicadas, Universidad Autonoma del Estado de Morelos, Avenida Universidad 1001, Col. Chamilpa, Cuernavaca 62209, Mexico; alfredo.brito@uaem.edu.mx (A.B.-F.); karengalvex@gmail.com (A.K.G.-L.); 3 Centro de Investigación Químicas, Universidad Autónoma del Estado de Morelos, Avenida Universidad 1001, Col. Chamilpa, Cuernavaca 62209, Mexico; hsaldarriaga@uaem.mx; 4 Departamento de Ingeniería Química y Metalurgia, Universidad de Sonora, Hermosillo 83000, Mexico

**Keywords:** copper, corrosion, green corrosion inhibitor, Furosemide

## Abstract

Copper and its alloys are extensively employed in a broad range of industrial applications owing to their outstanding mechanical, electrical, and thermal properties. However, their susceptibility to corrosion in aggressive environments remains a major challenge, making corrosion inhibitors one of the most practical and cost-effective strategies for extending their service life. Nevertheless, conventional synthetic inhibitors are often limited by their high cost and adverse environmental and health impacts resulting from their toxicity. In this context, the present work provides a comprehensive experimental and theoretical assessment of the corrosion inhibition performance of Furosemide as an environmentally friendly inhibitor for copper in 3.5 wt.% NaCl solution. The corrosion inhibition performance was evaluated experimentally through gravimetric measurements, potentiodynamic polarization, and electrochemical impedance spectroscopy (EIS), while the adsorption behavior of Furosemide was investigated using density functional theory (DFT) calculations. The results demonstrated that expired Furosemide effectively reduced the corrosion rate of copper, with the inhibition efficiency increasing as the inhibitor concentration increased and decreased with increasing temperature. A maximum inhibition efficiency of 90% was achieved at an inhibitor concentration of 400 ppm. The calculated Gibbs free energy of adsorption indicated that Furosemide adsorbs onto the copper surface through a mixed physisorption–chemisorption mechanism, following the Langmuir adsorption isotherm. Potentiodynamic polarization measurements further revealed that Furosemide predominantly suppresses the anodic dissolution reaction, indicating that it behaves as an anodic-type corrosion inhibitor. In addition, the presence of Furosemide significantly decreased the passive current density and shifted the breakdown potential toward more positive values, demonstrating an enhancement in the stability and protective character of the passive film. Electrochemical impedance spectroscopy showed that the corrosion process was governed by diffusion-controlled kinetics in the uninhibited solution, whereas the addition of Furosemide changed the corrosion mechanism to a charge-transfer-controlled process. Density functional theory (DFT) calculations provided additional insight into the inhibition mechanism of Furosemide. The calculated E_HOMO_) and E_LUMO_ values indicate that the molecule can both donate and accept electrons, reflecting its nucleophilic and electrophilic character and its strong affinity for adsorption on the copper surface. Furthermore, the relatively small energy gap (4.631 eV) suggests high molecular reactivity and facilitates electronic interactions with the metal surface. The estimated fraction of electrons transferred further supports the electron-donating ability of Furosemide during the adsorption process. Differences between the Fukui functions and the molecular electrostatic potential (MEP) maps are attributed to the distinct chemical information provided by each descriptor. Whereas the Fukui functions identify the most reactive atomic sites involved in soft donor–acceptor interactions, the MEP maps describe the molecular charge distribution governing electrostatic (hard–hard) interactions.

## 1. Introduction

Copper and its alloys are among the most extensively used engineering materials due to their excellent corrosion resistance, high electrical and thermal conductivity, and outstanding ductility and machinability. These properties have led to their widespread application in electrical wiring and cables, heat exchangers, marine equipment, pipelines, submarines, seawater desalination plants, roofing systems, architectural structures, and numerous other industrial sectors [1,2,3,4,5]. Upon exposure to atmospheric conditions, copper spontaneously develops a thin oxide film that provides a certain degree of corrosion protection. However, in aggressive environments containing chloride ions, acidic species, or other corrosive agents, this passive film may become unstable or locally break down, increasing the susceptibility of copper to corrosion and consequently impairing its structural integrity and functional performance [1,2,3,4]. Given the extensive use of copper in critical industrial applications, corrosion-related degradation represents a major technical and economic challenge, resulting in substantial maintenance, repair, and replacement costs.

Several strategies have been developed to mitigate metallic corrosion, including the application of protective coatings, cathodic protection, and alloy design optimization. Among these, the use of corrosion inhibitors remains one of the most practical, cost-effective, and widely implemented approaches. Conventional inhibitors, such as amines, amides, imidazolines, and azoles [5,6,7,8,9,10,11,12,13], exhibit high inhibition efficiencies owing to the presence of heterocyclic structures containing π-electron systems, polar functional groups, and multiple adsorption centers [14]. In particular, heteroatoms such as nitrogen, oxygen, sulfur, and phosphorus promote strong interactions with the metal surface through electron donation and/or electrostatic interactions, leading to the formation of protective adsorbed films that suppress both anodic and cathodic reactions [15]. Despite their excellent performance, many commercially available inhibitors are associated with high cost, poor biodegradability, and potential risks to human health and the environment. Consequently, increasing attention has been devoted to the development of sustainable corrosion inhibitors derived from environmentally benign sources, including plant extracts, amino acids, carbon-based nanomaterials, and expired pharmaceutical compounds [16,17,18,19,20,21,22,23,24,25,26].

Because many copper-based components, including marine structures, pipelines, submarines, and seawater desalination systems, operate in chloride-containing environments, numerous studies have explored the use of expired pharmaceuticals as corrosion inhibitors for copper and its alloys in NaCl media [24,25,26,27,28,29,30]. Varvara et al. [24] investigated expired Fluimucil (N-acetylcysteine) as a corrosion inhibitor for bronze in 3.5 wt.% NaCl using potentiodynamic polarization and electrochemical impedance spectroscopy (EIS). The inhibition efficiency increased with inhibitor concentration, reaching 83% at 36 mM. The protective effect was attributed to spontaneous adsorption of the inhibitor following the Langmuir adsorption isotherm through a mixed physicochemical adsorption mechanism, while the compound behaved as a mixed-type inhibitor. Fawzy et al. [25] evaluated expired streptomycin and neomycin as corrosion inhibitors for brass in NaCl solutions using weight-loss measurements, potentiodynamic polarization, and EIS. Maximum inhibition efficiencies of 88% and 91% were obtained at 500 ppm for streptomycin and neomycin, respectively. The inhibition efficiency decreased with increasing NaCl concentration and temperature, whereas both compounds acted as mixed-type inhibitors with a more pronounced influence on the anodic reaction. Their adsorption behavior was satisfactorily described by the Langmuir isotherm and was predominantly governed by physical adsorption. Wang et al. [27] reported that expired Domperidone effectively inhibited copper corrosion in 3.5 wt.% NaCl, reaching a maximum inhibition efficiency of 94% at 25 mg L^−1^. Electrochemical results indicated that Domperidone behaves as an anodic-type inhibitor, and its adsorption on the copper surface also followed the Langmuir isotherm. Likewise, Tasić et al. [30] demonstrated that expired Azithromycin is a highly efficient corrosion inhibitor for copper in 0.9 M NaCl. Using open-circuit potential measurements, potentiodynamic polarization, EIS, and scanning electron microscopy, they found that the inhibition efficiency increased with concentration, reaching 95% at 8.0 × 10^−3^ M. The inhibitor exhibited mixed-type behavior and was chemically adsorbed onto the copper surface according to the Langmuir adsorption model.

**Figure 1:**
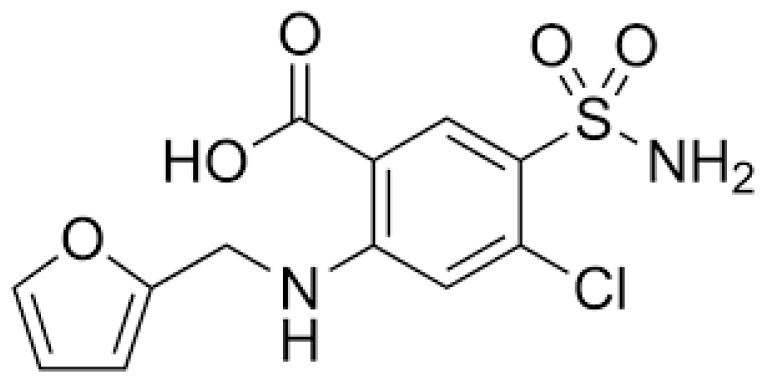
Chemical structure of Furosemide drug.

Furosemide (4-chloro-*N*-furfuryl-5-sulfamoylanthranilic acid) is a loop diuretic widely prescribed for the treatment of edema associated with congestive heart failure, hepatic cirrhosis, renal disorders, and hypertension [31]. As illustrated in Figure 1, its molecular structure consists of an aromatic benzene ring substituted with a carboxylic acid (–COOH), a sulfonamide group (–SO_2_NH_2_), and a chlorine atom, while a secondary amine (–NH–) links the benzene ring to a furan heterocycle containing an oxygen atom. The presence of multiple heteroatoms (N, O, S, and Cl), aromatic π-electron systems, and several electron-rich functional groups provides numerous potential adsorption centers capable of interacting with vacant orbitals of the metal surface, suggesting that Furosemide may exhibit excellent corrosion inhibition properties [31]. However, despite these favorable structural characteristics, its corrosion inhibition performance for copper in chloride-containing media has not yet been systematically investigated.

Accordingly, the present work aims to evaluate the effectiveness of expired Furosemide as a green corrosion inhibitor for copper in 3.5 wt.% NaCl solution. The inhibition performance was assessed by gravimetric measurements, potentiodynamic polarization, and electrochemical impedance spectroscopy. In addition, density functional theory (DFT) calculations were performed to elucidate the adsorption mechanism and to establish correlations between the electronic structure of Furosemide and its experimentally observed inhibition performance.

## 2. Materials and Methods

### 2.1. Testing Material

The commercial copper employed in this study had the following chemical composition (wt.%): Cu–0.25 Si–0.09 Fe. The material was supplied as cylindrical bars with a diameter of 10 mm. The test electrolyte consisted of a 3.5 wt.% NaCl solution prepared from analytical-grade sodium chloride (Sigma-Aldrich, St. Louis, MO, USA) and bidistilled water. A 1000 ppm stock solution of expired Furosemide (Ultra Laboratories, Guadalajara, Jalisco, Mexico) was prepared by dissolving an appropriate amount of the drug in bidistilled water. Test solutions containing 100, 200, 300, and 400 ppm of Furosemide were subsequently prepared by dilution of the stock solution with the electrolyte.

### 2.2. Weight-Loss Measurements

Weight-loss measurements were performed using cylindrical copper specimens with a diameter of 10 mm. Before each experiment, the specimens were mechanically polished with 600-grit SiC abrasive paper, thoroughly rinsed with bidistilled water, degreased with acetone (Baker, Phillipsburg, NJ, USA), dried in a stream of warm air, and accurately weighed.

The specimens were immersed in 100 mL of 3.5 wt.% NaCl solution in the absence and presence of different concentrations of Furosemide. The immersion tests were conducted for 72 h at 25, 40, and 60 °C in a thermostatically controlled water bath with a temperature stability of ±0.1 °C.

After exposure, the specimens were removed from the solution and the corrosion products were eliminated using a cleaning solution containing 5 wt.% H_2_SO_4_ (Baker) and 2.5 g L^−1^ benzotriazole (Sigma-Aldrich). The samples were then rinsed with bidistilled water, dried under warm air, and reweighed to determine the mass loss (Δ*M*). All measurements were performed in triplicate, and the average values were used for subsequent analysis. The inhibition efficiency (*IE*) was calculated according to the following equation:

(1) $$IE\left(\%\right)=\frac{\left({\Delta M}_{0}-{\Delta M}_{1}\right)}{{\Delta M}_{0}}100,$$

where Δ*M*_0_ and Δ*M*_1_ denote the mass losses measured in the absence and presence of the inhibitor, respectively. The inhibition efficiency (*η*_w_) was calculated from the weight-loss data according to the following expression:

(2) $$\eta_{w}=\frac{\left({\Delta M}_{0}-{\Delta M}_{1}\right)}{{\Delta M}_{0}},$$

The corrosion rate (*CR*, mg cm^−2^ h^−1^) was determined from the weight-loss measurements using the following expression:

(3) $$CR=\frac{\Delta M}{At},$$

where Δ*M* is the average mass loss (mg), *A* is the exposed surface area of the specimen (cm^2^), and *t* is the immersion time (h). The morphology of the corroded surfaces was examined using a JEOL scanning electron microscope (SEM) (JEOL, Tokyo, Japan).

### 2.3. Electrochemical Techniques

Open-circuit potential (OCP), potentiodynamic polarization, and electrochemical impedance spectroscopy (EIS) measurements were performed to evaluate the electrochemical behavior of copper in the absence and presence of Furosemide. All electrochemical experiments were carried out in a conventional three-electrode glass cell using a saturated Ag/AgCl electrode as the reference electrode, a graphite rod as the counter electrode, and a copper working electrode consisting of a 10 mm long cylindrical specimen embedded in epoxy resin, leaving a circular cross-sectional area exposed to the electrolyte. Measurements were conducted at 25 ± 1 °C using a Gamry Interface 1010 potentiostat (Gamry Instruments Inc., Warminster, PA, USA).

The OCP was monitored for 3600 s to allow stabilization of the electrode/electrolyte interface prior to electrochemical testing. Potentiodynamic polarization measurements were initiated from a potential 1200 mV below the corrosion potential (E_corr_) and scanned anodically at a rate of 1.0 mV s^−1^ up to 700 mV above E_corr_. The corrosion current density (I_corr_) was determined by Tafel extrapolation of the polarization curves.

Electrochemical impedance spectroscopy measurements were carried out at the stabilized OCP by applying a sinusoidal voltage perturbation with an amplitude of ±15 mV over a frequency range from 30 kHz to 0.1 Hz.

### 2.4. Quantum Chemical Calculations

To correlate the electronic properties of Furosemide with its corrosion inhibition performance, density functional theory (DFT) calculations were performed. All calculations were carried out using the RB3LYP/3-21G level of theory in conjunction with the integral equation formalism polarizable continuum model (IEFPCM), considering water as the solvent. The energies of the highest occupied molecular orbital E_HOMO_ and the lowest unoccupied molecular orbital E_LUMO_ were used to determine the molecular descriptors commonly employed to describe the adsorption behavior and chemical reactivity of corrosion inhibitors. These descriptors include the energy gap Δ*E*_gap_, ionization potential (I), electron affinity (*A*), electronegativity (χ), global hardness (η), global softness (σ), electrophilicity index (ω) and nucleophilicity index (ε). The corresponding parameters were calculated according to Equations (4)–(11) [32,33]:

(4) $$E_{gap}=E_{LUMO}-E_{HOMO},$$

(5) $$A=-E_{LUMO},$$

(6) $$I=-E_{HOMO},$$

(7) $$\chi =-Cp=\frac{I+A}{2},$$

(8) $$\eta =\frac{I-A}{2},$$

(9) $$\sigma =\frac{1}{\eta },$$

(10) $$\omega =\frac{\chi^{2}}{2\eta },$$

(11) $$\varepsilon =\frac{1}{\omega },$$

The number of electrons transferred (Δ*N*) is estimated using the following equation [34]:

(12) $$\Delta N=\frac{\chi_{Cu}-\chi_{inh}}{2(\eta_{Cu}+\eta_{inh})},$$

where $\chi_{Cu}$ and $\chi_{inh}$ represent the electronegativity of the metal surface and the expired drug acting as the corrosion inhibitor, respectively. Similarly, $\eta_{Cu}$ and $\eta_{inh}$ correspond to the chemical hardness of the metal surface and the inhibitor molecule. The theoretical values of $\chi_{Cu}$ and $\eta_{Cu}$ (4.96 eV and 0 eV, respectively) were used to estimate ΔN [32,33].

The Fukui functions, which are used to assess whether a molecule is more susceptible to nucleophilic ($f_{k}^{+}$) or electrophilic ($f_{k}^{-}$) attack, were calculated using Equations (13) and (14). Higher values of $f_{k}^{+}$ and $f_{k}^{-}$ indicate a greater tendency of a given atom to undergo nucleophilic or electrophilic attack, respectively, as reported in the literature [34,35].

(13) $$f_{k}^{+}=qk\left(N+1\right)-qk\left(N\right),$$

(14) $$f_{k}^{-}=qk\left(N\right)-qk\left(N-1\right),$$

where qk(N+1), qk(N−1), and qk(N) correspond to the anionic, neutral, and cationic Mulliken atomic charges. The Fukui dual function ($\Delta f_{k}$) serves as a quantitative descriptor of local reactivity within a molecule, where atoms with values greater than zero are considered electrophilic, while those with values below zero are regarded as nucleophilic [35]:

(15) $$\Delta f_{k}=f_{k}^{+}-f_{k}^{-},$$

## 3. Results and Discussion

### 3.1. Weight Loss Tests

**Figure 2:**
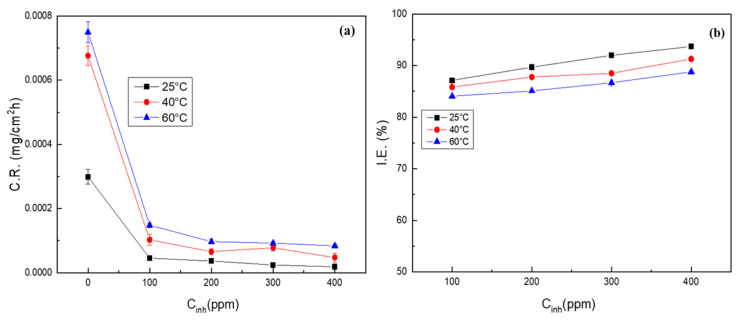
Effect of Furosemide concentration and testing temperature on the (**a**) Corrosion rate and (**b**) Inhibitor efficiency for Cu immersed in 3.5% NaCl solution.

The variation in corrosion rate and inhibition efficiency, as determined from weight loss measurements, as a function of Furosemide concentration at different temperatures in a 3.5 wt.% NaCl solution is shown in Figure 2.

As observed, the corrosion rate decreases, while the inhibition efficiency increases, with increasing the Furosemide concentration. In contrast, increasing the testing temperature leads to a higher corrosion rate and a corresponding reduction in inhibition efficiency. At 60 °C, the corrosion rate decreased from 7.5 × 10^−3^ mg·cm^−2^·h^−1^ in the uninhibited solution to 0.85 × 10^−3^ mg·cm^−2^·h^−1^ in the presence of 400 ppm of Furosemide, yielding a maximum inhibition efficiency of 88.7%. This increase can be due to an increase in the inhibitor concentration because of the adsorption of Furosemide molecules onto the copper surface, covering a bigger surface area and to the protection of metal due to the establishment of a corrosion products layer that isolates the metal from the aggressive chloride environment [24,25,26,27,28,29,30]. Comparable results have been reported in the literature. Fawzy et al. [25] reported maximum inhibition efficiencies of 87% and 90% using 400 ppm of expired Streptomycin and Neomycin, respectively, at 25 °C; however, these values decreased to 70% and 72% when the temperature increased to 60 °C. Similarly, Wang et al. [27] reported a maximum inhibition efficiency of 90% using 25 ppm of domperidone. Toghan et al. [36] investigated expired Moxifloxacin and Norfloxacin as corrosion inhibitors for copper in saline environments, obtaining inhibition efficiencies of 88% and 82%, respectively, at a concentration of 400 ppm. On the other side, Furosemide drug was investigated as corrosion inhibitor by El Maksoud et al. [34] for carbon steel in 1.0 M Hydrochloric acid, finding that the inhibitor efficiency increased with its concentration, reaching a maximum value of 83.2% with the addition of 300 ppm. Just for comparison, in this work, an inhibitor efficiency of 90% was obtained with the addition of 300 ppm of Furosemide at 25 °C. Therefore, the inhibition efficiencies obtained in the present study are consistent with and comparable to those reported in the literature, further confirming the effectiveness of expired pharmaceutical compounds as corrosion inhibitors for copper in chloride-containing environments.

### 3.2. Adsorption Isotherm

**Figure 3:**
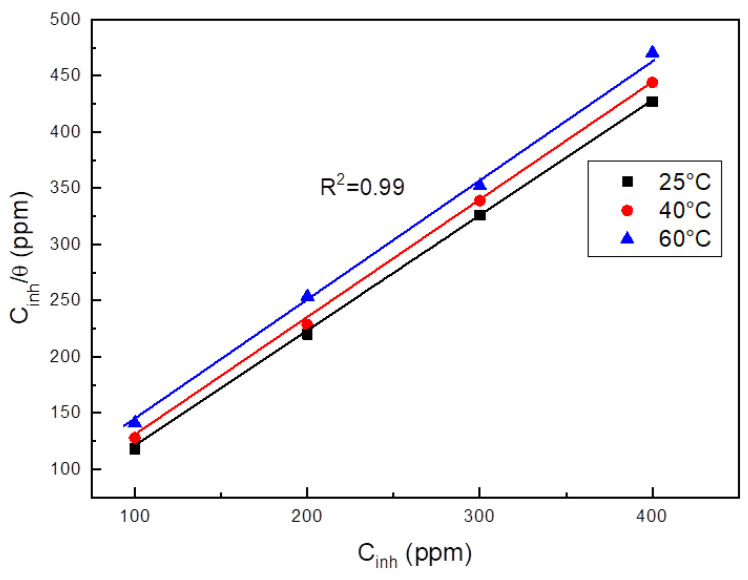
Langmuir adsorption isotherm for Cu in 3.5% NaCl solution in presence of Furosemide.

The reduction in the corrosion rate of copper in NaCl solution in the presence of Furosemide is attributed to the adsorption of inhibitor molecules onto the metal surface, resulting in the formation of a protective adsorbed film that isolates the substrate from the aggressive electrolyte. Therefore, elucidating the adsorption behavior of Furosemide is essential for understanding its inhibition mechanism. To characterize the adsorption process, the experimental data were analyzed using several adsorption isotherm models, including the Langmuir, Temkin, Frumkin, Flory–Huggins, and Freundlich isotherms. As illustrated in Figure 3, the Langmuir isotherm provided the best correlation with the experimental results, indicating that the adsorption process is adequately described by this model. The Langmuir adsorption isotherm is expressed as follows [36]:

(16) $$\frac{C_{inh}}{\theta }=\frac{1}{K_{ads}}+C_{inh},$$

In Equation (16) C_inh_ stands for the inhibitor concentration, θ is the metal surface area covered by the inhibitor, and K_ads_ is the adsorption constant which can be calculated by the intercept in Figure 3.

### 3.3. Thermodynamic Parameters

Calculation of the standard free energy, ${\Delta G}_{ads}^{0}$ was calculated as follows [37]:

(17) $${\Delta G}_{ads}^{0}=-RT\;In\;(10^{6}K_{ads}),$$

where T stands for the absolute temperature, R the universal gas constant and 10^6^ is the concentration of water in the solution. Obtained results for K_ads_ at 25, 40 and 60 °C were 0.054, 0.073 and 0.173 ppm^−1^ whereas computed values for ${\Delta G}_{ads}^{0}$ were −33.4, −29.1 and −27.0 kJ mol^−1^ respectively, which represents a mixed, physicochemical type of adsorption [36,37]. El Maksoud [34] obtained values for ${\Delta G}_{ads}^{0}$ between −41 and −49 kJ mol^−1^ for Cu in HCl implying a strong chemical type of adsorption. The negative value for the standard free energy denotes that the adsorption process is spontaneous.

**Figure 4:**
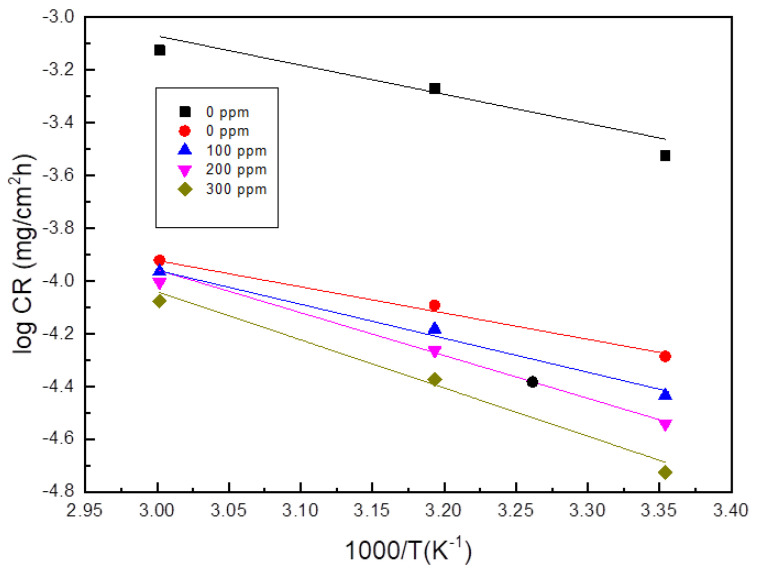
Arrhenius plot for log CR versus 1000/T for Cu in 3.5% NaCl solution in presence of Furosemide.

The apparent activation energy for Furosimede, E_a_, can be calculated according to following expression [38]:

(18) $$\log\left(CR\right)=A-\frac{E_{a}}{2.303RT},$$

where R is the universal gas constant, A represents the Arrhenius pre-exponential factor, and T is the absolute temperature. From the data presented in Figure 4, the linear relationship obtained by plotting log(CR) as a function of 1/T confirms the validity of the Arrhenius equation, and the activation energy was calculated from the slope of the resulting straight line.

**Figure 5:**
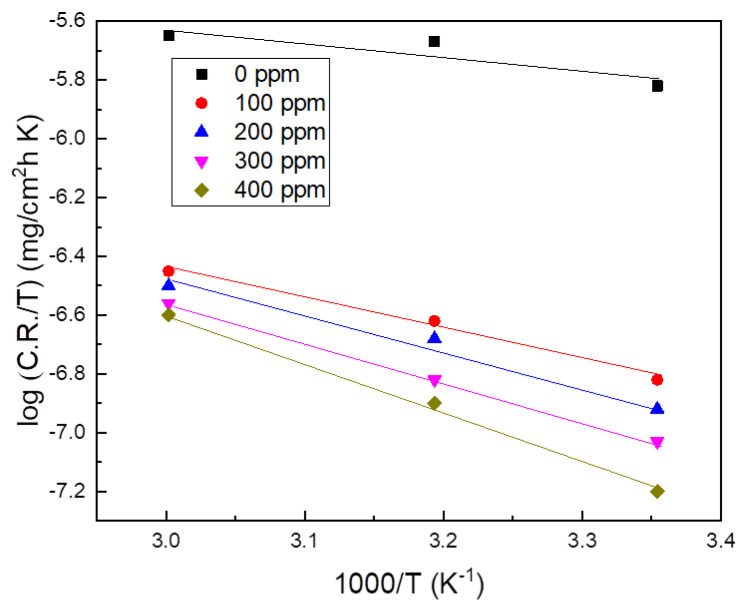
Log CR/T versus 1000/T for Cu in 3.5% NaCl solution in presence of Furosemide.

In addition, the enthalpy and entropy values (ΔH* and ΔS*, respectively) can be calculated using the Eyring transition state equation [39]:

(19) $$CR=\frac{RT}{Nh}e^{\frac{{\Delta S}^{*}}{R}}e^{\frac{{-\Delta H}^{*}}{RT}},$$

where N is the Avogadro’s number and h is the Plank-Boltzmann constant. Slope and intercept obtained from the log(CR/T) versus (1000/T) plots from Figure 5 were employed to calculate ΔS* and ΔH*.

Table 1 summarizes the calculated values of the activation energy E_a_, activation enthalpy (ΔH*), and activation entropy (ΔS*). As shown, the E_a_ values are consistently higher in the presence of Furosemide than in the uninhibited solution, indicating that the inhibitor increases the energy barrier for the corrosion reaction and, consequently, retards the corrosion process [38,39]. This increase in activation energy is consistent with the formation of an adsorbed protective film that hinders charge transfer at the metal/electrolyte interface. The positive values of ΔH*, indicate that the corrosion process is endothermic. In addition, ΔS* increases with increasing Furosemide concentration, suggesting significant changes in the interfacial structure during the adsorption process. In the absence of the inhibitor, water molecules are preferentially adsorbed on the copper surface in a relatively ordered arrangement. Upon addition of Furosemide, the adsorbed water molecules are progressively displaced by inhibitor molecules and released into the bulk solution, where they possess greater degrees of freedom. This replacement results in an increase in the disorder of the system, thereby giving rise to higher values of activation entropy [40,41].

### 3.4. Open Circuit Potential Measurements

**Figure 6:**
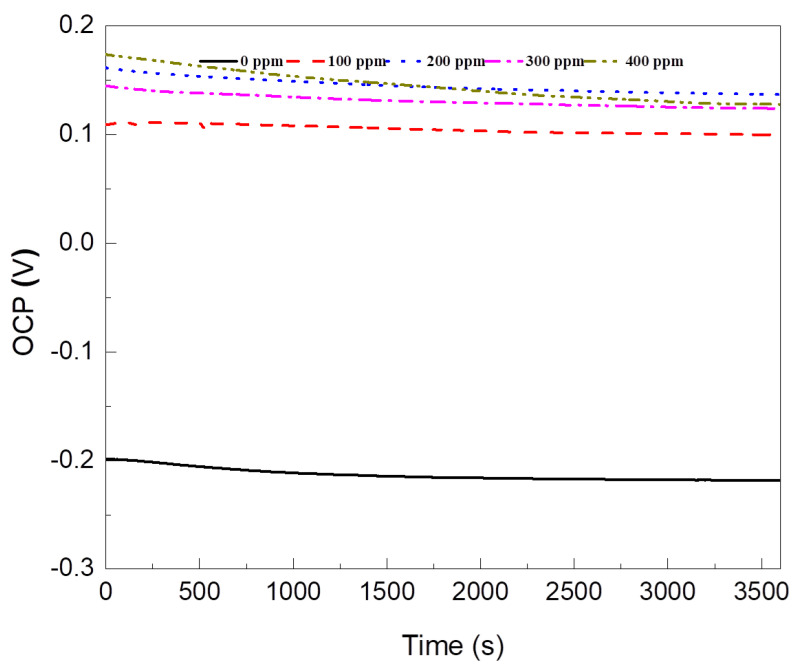
Variation on the OCP value for Cu in 3.5% NaCl solution in presence of Furosemide.

The variation in the open-circuit potential (OCP) of copper immersed in 3.5 wt.% NaCl solution containing different concentrations of Furosemide is shown in Figure 6. In the uninhibited solution, the OCP remained relatively stable at approximately −0.20 V throughout the immersion period. This behavior is attributed to the formation of a surface film composed of copper oxides, mainly Cu_2_O and CuO, together with basic copper chloride corrosion products such as Cu_2_(OH)_3_Cl and Cu_4_(OH)_6_Cl_2_, which provide partial protection to the metal surface [42]. The addition of Furosemide shifted the OCP toward more positive (nobler) potentials, with the potential stabilizing between approximately 0.10 and 0.20 V depending on the inhibitor concentration. This positive displacement indicates the adsorption of Furosemide molecules onto the copper surface, resulting in the formation of a protective adsorbed film that modifies the metal/electrolyte interface and suppresses the corrosion reactions. The shift toward nobler potentials also suggests that the inhibitor predominantly influences the anodic dissolution process, although both anodic and cathodic reactions may be affected by the adsorption of the inhibitor molecules [10,11,12] and that the inhibitor completely suppresses the anodic sites, behaving as an anodic type of inhibitor.

### 3.5. Potentiodynamic Polarization Curves

**Figure 7:**
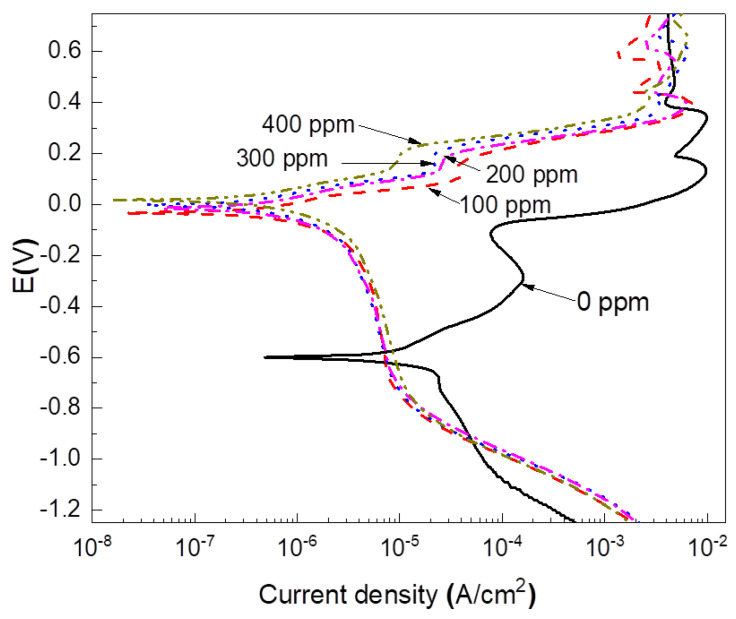
Effect of Furosemide concentration on the polarization curves for Cu immersed in 3.5% NaCl solution.

The effect of Furosemide concentration on the polarization curves of copper in a 3.5% NaCl solution is shown in Figure 7.

In the absence of the inhibitor, the curve displays an active–passive behavior, with a passive region between −0.2 and −0.1 V which disappear when Furosemide is added because, as some authors have pointed out, copper does not have effective passive film in saltwater environment and what looks like a passive region between −0.2 and −0.1 V is due to the chemical evolution of the copper from Cu^1+^ to Cu^2+^ [43]. Upon the addition of Furosemide, the corrosion potential (E_corr_) shifts toward more noble values. The corrosion potential shifted progressively toward more positive values, from approximately −600 mV in the uninhibited solution to 20 mV in the presence of 400 ppm Furosemide, indicating the formation of a protective film on the copper surface. Furthermore, the polarization curves obtained in the inhibited solutions exhibited a well-defined passive region between 0.2 and 0.3 V. As the Furosemide concentration increased, the passive current density decreased, whereas the breakdown (pitting) potential shifted toward more positive values, demonstrating an improvement in the stability and protectiveness of the passive film. As summarized in Table 2, the addition of Furosemide modified both the anodic and cathodic Tafel slopes, with a more pronounced effect on the anodic branch. This behavior indicates that Furosemide acts as an anodic-type inhibitor. The observed inhibition mechanism is attributed to the adsorption of Furosemide molecules and the consequent formation of a protective surface film, which suppresses both the anodic dissolution of copper and the cathodic reduction reaction, thereby reducing the overall corrosion rate [44,45,46].

In NaCl solution, Furosemide may exist in both neutral and protonated forms, and its adsorption on the copper surface is expected to proceed through multiple mechanisms. The neutral molecules can be chemisorbed via donor–acceptor interactions involving the π-electrons of the aromatic and heterocyclic rings, as well as the lone-pair electrons of the O, N, and S atoms, with the vacant *d* orbitals of surface Cu atoms [38]. These interactions promote the formation of coordinated adsorption bonds, enhancing surface coverage. The adsorption of the protonated species follows a different pathway. Since the copper surface is positively charged under near-neutral conditions, direct adsorption of protonated Furosemide is hindered by electrostatic repulsion. However, chloride ions from the electrolyte are specifically adsorbed onto the copper surface, producing a negatively charged interfacial layer. This adsorbed chloride layer facilitates the electrostatic attraction of the protonated inhibitor, leading to the formation of ion-pair complexes at the metal/solution interface. Consequently, the adsorption process involves a combination of chemisorption by the neutral species and electrostatic interactions mediated by specifically adsorbed chloride ions for the protonated species. The resulting adsorbed film acts as an effective barrier, suppressing both the anodic dissolution of copper and the associated cathodic reactions, thereby reducing the overall corrosion rate.

The addition of Furosemide reduced both the anodic and cathodic current densities, resulting in a marked decrease in the corrosion current density (I_corr_), as summarized in Table 2. The I_corr_ value decreased by approximately one order of magnitude, from 2.0 × 10^−5^ mA cm^−2^ in the uninhibited solution to 2.0 × 10^−6^ mA cm^−2^ in the presence of 400 ppm of Furosemide, corresponding to the highest inhibition efficiency of 90%.

The inhibition performance of Furosemide compares favorably with that of other expired pharmaceutical compounds reported in the literature for copper-based alloys exposed to chloride media. Varvara et al. [24] reported a maximum inhibition efficiency of 83% for expired Fluimucil on bronze in 3.5 wt.% NaCl, with a minimum I_corr_ of 3.4 × 10^−4^ mA cm^−2^ at an inhibitor concentration of 36 mM. Similarly, Toghan et al. [36] investigated expired Moxifloxacin and Norfloxacin as corrosion inhibitors for copper in 3.5 wt.% NaCl and obtained minimum I_corr_ values of 9.2 × 10^−4^ mA cm^−2^ at an inhibitor concentration of 500 ppm, corresponding to a maximum inhibition efficiency of 85%. Fawzy et al. [25] evaluated expired streptomycin and neomycin under similar conditions and reported inhibition efficiencies of 87% and 91%, respectively, at 500 ppm. These efficiencies were associated with the lowest corrosion current densities reported for each inhibitor. Overall, the present results demonstrate that expired Furosemide provides corrosion protection comparable to, or better than, that of several expired pharmaceutical inhibitors previously investigated for copper in chloride-containing environments. In contrast, El Maksoud [34] did not observe passive behavior for copper in HCl solution, either in the absence or presence of Furosemide. Under these conditions, Furosemide inhibited only the anodic dissolution of copper, while the cathodic oxygen reduction and hydrogen evolution reactions remained essentially unaffected.

It should be noticed that the inhibitor efficiency values in the gravimetric tests are slightly different to those obtained in the potentiodynamic polarization tests. The difference between the theoretical and experimental CR can be due to the fact that the weight loss testing has some errors, as the corrosion products do not seem to be removed completely. The CR calculated using corrosion current of the potentiodynamic polarization curve can be more accurate [47].

### 3.6. Electrochemical Impedance Spectroscopy Tests

**Figure 8:**
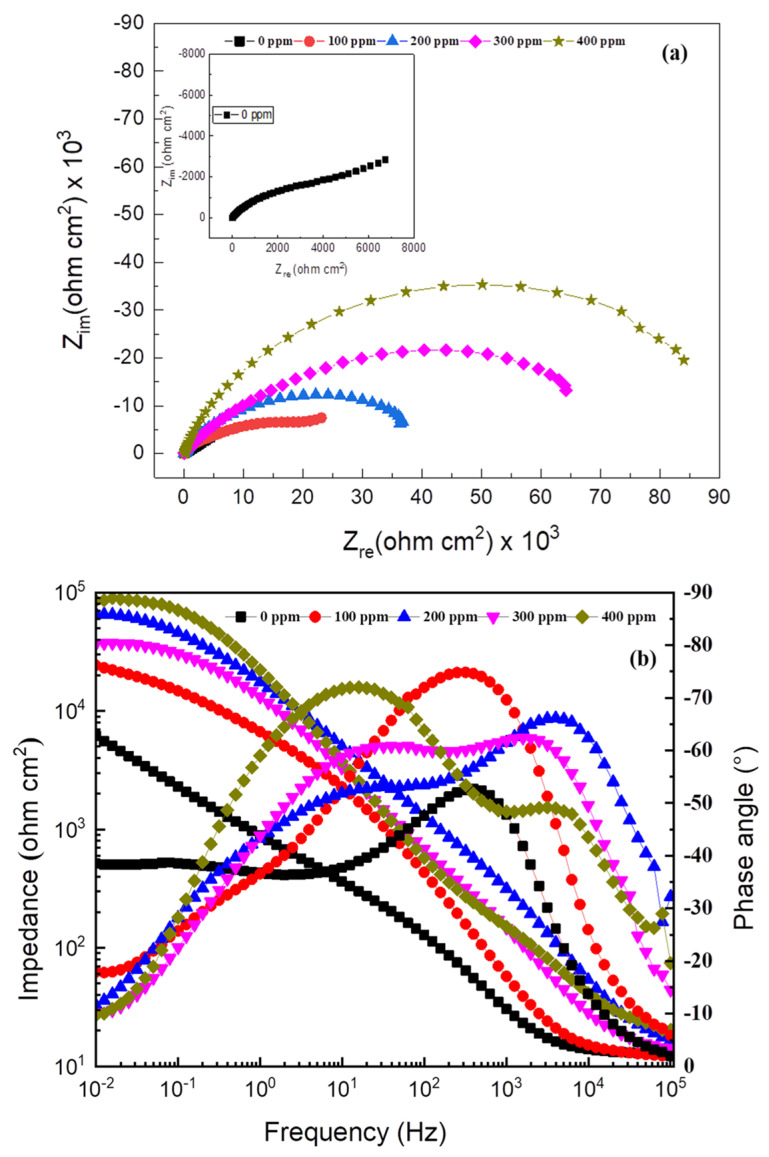
Effect of Furosemide concentration on the (**a**) Nyquist and (**b**) Bode plots Cu immersed in 3.5% NaCl solution.

To further elucidate the interfacial processes governing copper corrosion in the absence and presence of Furosemide, the electrochemical impedance spectroscopy (EIS) results are presented in Figure 8 in the form of Nyquist and Bode plots.

The Nyquist spectrum obtained for copper in the uninhibited 3.5 wt.% NaCl solution (Figure 8a) consists of a depressed capacitive semicircle in the high- and intermediate-frequency regions, followed by a straight line in the low-frequency region, characteristic of Warburg impedance. This response indicates that the corrosion process is partially controlled by mass transport, which may be associated with the diffusion of Cu^2+^ ions from the metal surface into the bulk solution and/or the diffusion of dissolved oxygen toward the electrode surface [48]. The addition of 100 ppm Furosemide produced only minor changes in the impedance response, and the Warburg feature remained evident. However, at inhibitor concentrations of 200 ppm and above, the low-frequency diffusion tail disappeared and the Nyquist plots exhibited a single depressed capacitive loop, indicating that the corrosion process became predominantly controlled by charge-transfer kinetics. A similar transition has been reported by El Maksoud et al. [34] for copper in HCl containing expired Furosemide. Moreover, the diameter of the capacitive loop increased progressively with increasing inhibitor concentration, reflecting a significant enhancement in the charge-transfer resistance and, consequently, the corrosion resistance of the copper surface.

The corresponding Bode plots (Figure 8b) further confirm the protective effect of Furosemide. The impedance modulus increased by approximately one order of magnitude at 400 ppm compared with the uninhibited solution, indicating a substantial improvement in the corrosion resistance. In addition, the phase-angle spectra exhibited a single time constant in the absence of the inhibitor, with a maximum phase angle of approximately −50°. In contrast, the inhibited solutions showed two distinguishable time constants and a maximum phase angle close to −75°, suggesting the formation of a compact and protective surface film associated with the adsorption of Furosemide in conjunction with the corrosion product layer.

**Figure 9:**
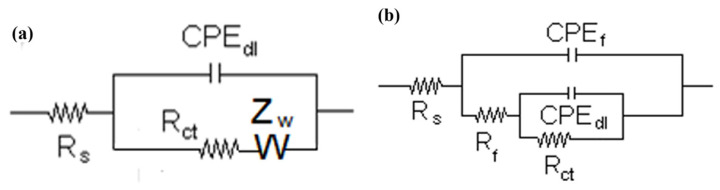
Electric circuits to fit the EIS data for Cu immersed in 3.5% NaCl solution containing (**a**) 0 and 100 ppm and (**b**) 200, 300 and 400 ppm of Furosemide [49].

The electrochemical impedance spectroscopy (EIS) data were fitted using the equivalent electrical circuits shown in Figure 9 [49]. In these circuits, R_s_ represents the solution resistance, R_ct_ the charge-transfer resistance, C_dl_ the double-layer capacitance, R_f_ the resistance of the surface film, C_f_ its capacitance, and R_w_ the resistance associated with the Warburg diffusion element.

In practice, real systems do not exhibit ideal behavior due to surface inhomogeneities, such as roughness and porosity. Therefore, ideal capacitors are replaced by constant phase elements (CPEs), whose impedance (Z_CPE_) is given by [43]:

(20) $$Z_{CPE}=Y^{-1}{\left(i\omega \right)}^{-n},$$

where the electrical admittance is represented by Y is, *i* is √−1, ω is 2π*f*, *f* the frequency and *n* have the meaning of a phase shift [43]. Alternatively, the double-layer capacitance C_dl_ was calculated using the following relationship [43]:

(21) $$C_{dl}=\left(\frac{\varepsilon \varepsilon_{0}}{\lambda }\right)A,$$

where ε_0_ denotes the vacuum permittivity, ε is the dielectric constant of the medium, λ represents the thickness of the electrochemical double layer, and (*A*) corresponds to the exposed electrode area. The electrochemical parameters obtained from the EIS fitting procedure are summarized in Table 3. The inhibition efficiency (IE) was calculated from the impedance data according to the following equation [48]:

(22) $$IE\left(\%\right)=\frac{R_{ct2}-R_{ct1}}{R_{ct2}}\times 100,$$

where the charge transfer resistance with and without inhibitor are denoted by $R_{ct2}$ and $R_{ct1}$ respectively. It should be noted that the diffusion resistance value, R_W_ for solution containing 0 and 100 ppm increase with the inhibitor concentration likely associated with the diffusion of Cu^2+^ ions from the metal surface into the bulk solution or the diffusion of dissolved oxygen from the bulk solution toward the metal surface [50]. A progressive increase in the R_ct_ value was observed with increasing Furosemide concentration, which can be attributed to the inhibition of charge transfer processes and the reduced dissolution of Cu^2+^ ions across the metal/electrolyte interface due to the lower corrosion rate in the presence of the inhibitor [46,47]. Conversely, the Y_dl_ parameter decreased as the inhibitor concentration increased. Since Y_dl_ is related to the double-layer capacitance C_dl_, its decrease can be explained by the increase in the thickness of the electrochemical double layer according to Equation (21) [50]. This behavior is associated with the displacement of adsorbed water molecules by Furosemide molecules, which possess a larger molecular size and occupy adsorption sites on the copper surface, leading to changes in the interfacial structure [51]. Furthermore, the increase in the corrosion product film resistance R_f_ with increasing Furosemide concentration suggests the development of a more compact and protective surface film, enhancing the barrier properties of the copper/electrolyte interface.

### 3.7. Surface Analysis

**Figure 10:**
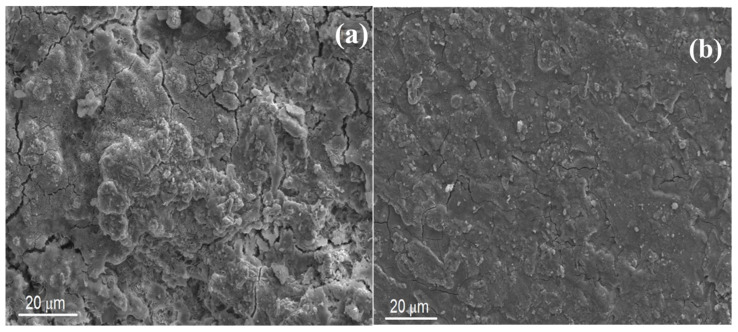
SEM micrographs of Cu corroded in 3.5% NaCl solution with the addition of (**a**) 0 and (**b**) 400 ppm of Furosemide.

The surface morphologies of copper specimens exposed to 3.5 wt.% NaCl solution in the absence and presence of Furosemide are presented in Figure 10. The specimen immersed in the uninhibited solution (Figure 10a) exhibits a non-uniform corrosion product film characterized by extensive cracking and a porous structure. These defects provide preferential pathways for electrolyte penetration, facilitating the localized attack of the underlying copper substrate. In contrast, the specimen exposed to 400 ppm expired Furosemide (Figure 10b) displays a more compact, homogeneous, and continuous surface film. Although some microcracks and micropores remain visible, their density is considerably lower than that observed in the uninhibited condition. The presence of these residual defects may still allow limited access of aggressive species to the metal surface, which could explain the inhibition efficiency below 100%. These defects are formed because inhibitor molecules adsorb progressively onto the metal surface, and complete monolayer coverage is seldom achieved. In addition to this, surface heterogeneities such as grain boundaries, inclusions, scratches, variations in local inhibitor concentration, crystallographic orientation, or surface energy produce regions with different film thicknesses, leaving microscopic pores or uncovered areas. As discussed above, the observed protection is attributed to the development of a surface corrosion product film containing copper oxides (CuO and Cu_2_O) and basic copper chloride compounds, such as Cu_2_(OH)_3_Cl and Cu_4_(OH)_6_Cl_2_, which contributes to reducing the interaction between the copper substrate and the aggressive electrolyte [42].

### 3.8. Theoretical Calculations

**Figure 11:**
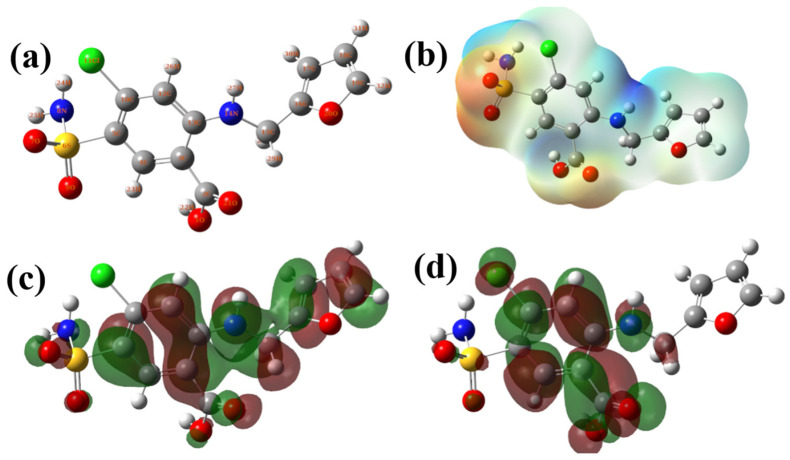
(**a**) Optimized structure, (**b**) MEP, (**c**) HOMO and (**d**) LUMO distributions for the expired Furosemide drug.

Figure 11 presents the Furosemide molecule, with its optimized structure shown in Figure 11a. The molecular electrostatic potential (MEP), illustrated in Figure 11b, displays a red region localized on the sulfonamide group (–SO_2_NH_2_) attached to the C5 atom of the benzene ring. This indicates that this functional group is electron-rich, with a high negative charge density, making it susceptible to electrophilic attack. Consequently, it can act as an electron donor to the copper surface, facilitating the adsorption of Furosemide onto the metal substrate. In contrast, the blue region, characterized by a lower electron density, is localized around the N14 atom bonded to the C15 atom of the benzene ring. This region is prone to nucleophilic attack and can act as an electron acceptor from the metal surface. The spatial distribution of the frontier molecular orbitals is presented in Figure 11c and Figure 11d, corresponding to the E_HOMO_ and E_LUMO_ regions, respectively.

The E_HOMO_ orbital exhibits a high electron density primarily localized on the –SO_2_NH_2_ group and the nitrogen atom, in agreement with the MEP results, indicating that these regions act as nucleophilic sites. Additionally, variations in the E_HOMO_ distribution are observed along the aromatic benzofuran ring system and around the chlorine atom. Table 4 lists the calculated E_HOMO_ value of −6.290 eV, which is consistent with values reported in the literature [31,32,33]. For example, imidazoline compounds have been reported to exhibit E_HOMO_ values of approximately −6.28 eV, while thiazoles and benzamides typically show values in the range of −5 to −7 eV [31,32,33]. Notably, these compounds also demonstrate good corrosion inhibition performance, particularly when E_HOMO_ values fall within the range of −6.0 to −6.5 eV.

The E_LUMO_ distribution (Figure 11c) shows a pronounced LUMO density over the benzene rings, indicating the presence of π* antibonding orbitals capable of accepting electrons. Additionally, the carboxylic acid group (–COOH) exhibits significant LUMO density, suggesting that it is an electron-deficient and reactive site. In contrast, the red/brown regions observed in Figure 11d correspond to areas of lower LUMO density, where the sulfonamide group (–SO_2_NH_2_) displays moderate LUMO character. The sulfur atom also presents some LUMO density, reflecting the electron-withdrawing nature of the SO_2_ group. Table 4 reports an E_LUMO_ value of −1.659 eV, which suggests that the low-energy vacant molecular orbitals of Furosemide can accept electrons from the copper surface, facilitating inhibitor–metal interactions. This value is consistent with those reported in the literature, e.g., Khabazi et al. [31] studied several tetrazoles and their derivates, finding E_LUMO_ values between −1.489 and −4.669 eV, reporting their effectiveness as corrosion inhibitor. Otherwise, Jafarzadegan et al. [48] tested the 4-amino-3-thioxo-6-methyl-1,2,4-triazine-5-one and its derivatives as corrosion inhibitor, reporting E_LUMO_ values between −2 and −6 eV which are lower than those for Furosemide. Furthermore, the ΔE_gap_ of the Furosemide reports a value of 4.631 eV, which based on the findings of Bourzi et al., who reported a ΔE_gap_ of 4.217 eV, suggests that the Furosemide is easily adsorbed on the metal surface [50]. The Δ*N* estimated is 0.2128 eV, suggesting that Furosemide acts as an electron donor. Moreover, ΔN < 3.6 confirming the electron-donating ability of the inhibitor, which is associated with its calculated chemical softness and hardness parameters [51,52,53].

The Fukui indices and Mulliken atomic charges calculated for Furosemide are presented in Table 5. According to these parameters, the highest $f_{k}^{-}$ value is associated with the N14 atom, which exhibits a dual descriptor ($\Delta f_{k}$) lower than zero, indicating that this site in the Furosemide molecule is highly susceptible to electrophilic attack. In contrast, the highest $f_{k}^{+}$ value corresponds to the O21 atom, with $\Delta f_{k}>0$, suggesting that this position is particularly prone to nucleophilic attack. The differences observed between the MEP and Fukui index results can be attributed to their distinct theoretical foundations: the Fukui function describes soft–soft interactions, whereas the MEP characterizes hard–hard interactions [53].

## 4. Conclusions

Expired Furosemide significantly reduced the corrosion rate of copper in a 3.5% NaCl solution, with the inhibition efficiency increasing with concentration and reaching a maximum value of 90% at 400 ppm but decreased with the testing temperature. The standard Gibbs free energy of adsorption (ΔG°_ads_) at 25, 40, and 60 °C was −33.4, −29.1, and −27.0 kJ·mol^−1^, respectively, indicating that Furosemide adsorbs onto the copper surface through a mixed physicochemical mechanism, consistent with the Langmuir adsorption isotherm. Furosemide affected mainly anodic dissolution electrochemical reactions behaving, thus as an anodic type of corrosion inhibitor. The addition of Furosemide reduced the passive current density while shifting the breakdown potential toward more positive values, indicating enhanced stability and protectiveness of the passive film. In the absence of the inhibitor, the corrosion process was predominantly controlled by diffusion. However, the presence of Furosemide changed the controlling mechanism to charge-transfer kinetics, as evidenced by the decrease in the double-layer capacitance (Cdl) and the concomitant increase in the charge-transfer resistance (R_ct_). These results indicate the adsorption of Furosemide molecules onto the copper surface, leading to the formation of a protective barrier that effectively hinders the corrosion process. Density functional theory (DFT) calculations provided further insight into the inhibitory behavior of Furosemide. The calculated E_HOMO_ and E_LUMO_ values indicate that the molecule exhibits both electron-donating and electron-accepting capabilities, reflecting its nucleophilic and electrophilic characteristics and its potential to interact with the metal surface. The calculated energy gap, ΔE_gap_, of 4.631 eV suggests sufficient molecular reactivity to facilitate adsorption onto the copper surface. Furthermore, the estimated fraction of electrons transferred, Δ*N*, supports the electron-donating ability of Furosemide during the adsorption process. The differences observed between the Fukui functions and the molecular electrostatic potential (MEP) maps arise from the different types of chemical information provided by these descriptors: the Fukui functions identify reactive sites involved in soft–soft interactions, whereas the MEP maps describe charge distribution associated with electrostatic (hard–hard) interactions.

## Figures and Tables

**Table 1:** Thermodynamic parameters for Cu immersed in 3.5% NaCl solution with the addition of Furosemide drug.

Cinh (ppm)	Ea kJ mol^−1^	ΔH* kJ mol^−1^	ΔS* J mol^−1^K^−1^
0	16.2	7.1	−32.1
100	19.1	8.0	−28.1
200	22.6	8.7	−22.7
300	31.1	12.3	−16.7
400	34.8	14.0	−12.4

**Table 2:** Electrochemical parameters for the polarization curves for Cu immersed in 3.5% NaCl solution with the addition of Furosemide drug.

C_inh_ (ppm)	E_corr_ (mV)	I_corr_ (mA/cm^2^)	β_a_ (mV/dec)	β_c_ (mV/dec)	R_p_ (ohm cm^2^)	I.E. (%)	θ
0	−600	2.0 × 10^−5^	170	660	6648	0	0
100	−30	6.0 × 10^−6^	80	600	30,827	70	0.7
200	−15	4.0 × 10^−6^	65	555	43,857	80	0.8
300	5	3.0 × 10^−6^	55	540	83,973	85	0.85
400	20	2.0 × 10^−6^	45	520	95,473	90	0.9

**Table 3:** Electrochemical parameters used to fit the EIS results for Cu in 3.5% NaCl without and with the addition of Furosemide.

C_inh_ (ppm)	χ^2^	R_ct_ (ohm cm^2^)	Y_dl_ (ohm^−1^ cm^−2^ s^n^)	n_ct_	R_W_ (ohm cm^2^)	R_f_ (ohm cm^2^)	Y_f_ (ohm^−1^ cm^−2^ s^n^)	n^f^	I.E. (%)
0	3.76 × 10^−5^	150	3.18 × 10^−5^	0.9	6404	-----	-----	----	----
100	1.54 × 10^−4^	550	6.06 × 10^−6^	0.9	30,227	-----	-----	----	---
200	8.11 × 10^−5^	790	3.03 × 10^−6^	0.9	-------	42,617	1.60 × 10^−5^	0.6	81
300	1.13 × 10^−4^	1150	1.31 × 10^−6^	0.9	-------	82,741	1.57 × 10^−5^	0.6	87
400	1.07 × 10^−4^	1500	8.26 × 10^−6^	0.7	-----	98,094	1.66 × 10^−6^	0.9	90

**Table 4:** DFT parameters for the Furosemide as corrosion inhibitor.

E_HOMO_ (eV)	E_LUMO_ (eV)	ΔE_gap_ (eV)	*A* (eV)	*I* (eV)	μ (eV)	χ (eV)	η (eV)	σ (eV^−1^)	Ω (eV)	ε (eV^−1^)	Δ*N*
−6.290	−1.659	4.631	1.659	6.290	−3.9745	3.9745	2.3155	0.432	3.411	0.293	0.2128

**Table 5:** Fukui Index and Mulliken atomic charge estimated for Furosemide.

Atom	qk (N − 1)	qk (N + 1)	qk (N)	$f_{k}^{-}$	$f_{k}^{+}$	$\Delta f_{k}$
1O	0.7421	0.675	−0.6952	0.0202	0.0469	0.0267
2C	0.6756	0.7705	0.7733	−0.0028	0.0977	0.0949
3C	−0.2892	−0.1361	−0.2223	0.0863	0.0669	−0.0194
4C	−0.2424	−0.1374	−0.1409	0.0035	0.1015	0.098
5C	−0.2681	−0.1741	−0.2674	0.0933	0.0007	−0.0926
6S	1.8275	1.835	1.8393	−0.0043	0.0117	0.0075
7O	−0.8742	−0.8251	−0.8516	0.0265	0.0227	−0.0038
8N	−0.9603	−0.9394	−0.9501	0.0107	0.0102	−0.0005
9O	−0.8798	−0.8427	−0.8629	0.0202	0.0168	−0.0034
10C	−0.0863	0.017	0.0239	−0.0069	0.1102	0.1033
11Cl	−0.1042	0.0221	−0.0183	0.0404	0.0859	0.0455
12C	−0.3477	−0.1603	−0.2475	0.0873	0.1002	0.0129
13C	0.2263	0.2449	0.2647	−0.0198	0.0384	0.0186
14N	−0.5798	−0.3642	−0.5399	0.1757	0.0399	−0.1358
15C	−0.2395	−0.2656	−0.2405	−0.0251	−0.001	−0.0241
16C	0.2863	0.3318	0.2762	0.0556	−0.0102	−0.0455
17C	−0.296	−0.2242	−0.2835	0.0593	0.0126	−0.0467
18C	−0.2961	−0.2643	−0.2939	0.0296	0.0022	−0.0274
19C	0.1019	0.2024	0.1119	0.0906	0.01	−0.0806
20	−0.4872	−0.472	−0.483	0.011	0.0042	−0.0068
21O	−0.7063	−0.5603	−0.5946	0.0343	0.1117	0.0774

## Data Availability

The original contributions presented in this study are included in the article. Further inquiries can be directed to the corresponding authors.
